# Description and Characterization of the *Odontella aurita* OAOSH22, a Marine Diatom Rich in Eicosapentaenoic Acid and Fucoxanthin, Isolated from Osan Harbor, Korea

**DOI:** 10.3390/md21110563

**Published:** 2023-10-27

**Authors:** Sung Min An, Kichul Cho, Eun Song Kim, Hyunji Ki, Grace Choi, Nam Seon Kang

**Affiliations:** Department of Microbial Resources, National Marine Biodiversity Institute of Korea, Seocheon 33662, Republic of Korea; sman@mabik.re.kr (S.M.A.); kichul.cho@mabik.re.kr (K.C.); kes2523@mabik.re.kr (E.S.K.); hki@mabik.re.kr (H.K.); gchoi@mabik.re.kr (G.C.)

**Keywords:** carotenoid pigments, fatty acids, *Odontella aurita*, culture condition, ultrastructure, 18S rDNA

## Abstract

Third-generation biomass production utilizing microalgae exhibits sustainable and environmentally friendly attributes, along with significant potential as a source of physiologically active compounds. However, the process of screening and localizing strains that are capable of producing high-value-added substances necessitates a significant amount of effort. In the present study, we have successfully isolated the indigenous marine diatom *Odontella aurita* OAOSH22 from the east coast of Korea. Afterwards, comprehensive analysis was conducted on its morphological, molecular, and biochemical characteristics. In addition, a series of experiments was conducted to analyze the effects of various environmental factors that should be considered during cultivation, such as water temperature, salinity, irradiance, and nutrients (particularly nitrate, silicate, phosphate, and iron). The morphological characteristics of the isolate were observed using optical and electron microscopes, and it exhibited features typical of *O. aurita*. Additionally, the molecular phylogenetic inference derived from the sequence of the small-subunit 18S rDNA confirmed the classification of the microalgal strain as *O. aurita*. This isolate has been confirmed to contain 7.1 mg g^−1^ dry cell weight (DCW) of fucoxanthin, a powerful antioxidant substance. In addition, this isolate contains 11.1 mg g^−1^ DCW of eicosapentaenoic acid (EPA), which is one of the nutritionally essential polyunsaturated fatty acids. Therefore, this indigenous isolate exhibits significant potential as a valuable source of bioactive substances for various bio-industrial applications.

## 1. Introduction

Microalgae contain various functional substances [1]. Unlike resources such as terrestrial plants and seaweeds, they can be cultured in large quantities, which has the advantage of securing raw materials in a stable manner [2]. Microalgae contain a significant amount of useful high-value substances, including unsaturated fatty acids (such as omega-3 fatty acids), natural pigments (such as astaxanthin, lutein, and fucoxanthin), and polysaccharides and oligosaccharides (such as fucoidan, alginic acid, and carrageenan) [3]. They are used in various bio-industries such as the food, health and functional food, cosmetics, and pharmaceutical industries [4]. However, despite the high diversity of microalgae, only a few, such as *Chlorella* (Chlorophyta) and *Spirulina* (Cyanobacteria), are used as raw food materials and functional ingredients [5]. Since the development of useful materials derived from microalgae has been limited to certain species, there is strong potential for discovering new sources for biomaterial development in the future.

Diatoms (Bacillariophyta) are the dominant group of microalgae in marine environments [6]. They are the primary producers in coastal ecosystems [7]. They play a significant role in the biogeochemical cycles of carbon and silicate [8]. Since diatoms have high nutritional value and industrial potential, numerous studies have been conducted on various diatom species, including *Cylindrotheca closterium*, *Nanofrustulum shiloi*, *Nitzschia laevis*, *Odontella aurita*, *Phaeodactylum tricornutum*, *Skeletonema costatum*, and *Thalassiosira weissflogii* [9,10,11,12,13,14,15]. Among the various species, *Odontella aurita* has garnered significant attention. This species is the type species of the genus *Odontella* and is classified under the order Eupodiscales. The size of this species exhibits significant variability, ranging from 10 to 100 μm [16]. Additionally, it has the ability to form colonies characterized by their ribbon-like shape [16]. This species exhibits a global distribution, being found in both benthic and planktonic forms [17]. It has been observed to occasionally form blooms during the winter and early spring [17]. This species is of significant interest due to its remarkable capacity to accumulate eicosapentaenoic acid (EPA) and fucoxanthin [18]. Polyunsaturated fatty acids (PUFAs), such as EPA, have been recognized for their ability to provide a variety of health benefits to individuals, and traditionally, these fatty acids have been primarily obtained from fish oil [19]. As awareness of animal welfare and the promotion of vegan culture continue to grow, microalgae are being recognized as a viable alternative to fish oil [20]. Among these microalgae, *O. aurita* stands out due to its high content of EPA, comprising approximately 25–26% of its total fatty acid composition [18]. Consequently, *O. aurita* is being explored as a promising source of EPA [21]. Fucoxanthin, a marine xanthophyll present in brown algae and diatoms, exhibits a diverse array of bioactivities, including anti-oxidant, anti-cancer, and anti-obesity properties [22]. Additionally, it has been reported that the substance contains a variety of beneficial components, including fiber, phytosterols, protein, and minerals [23,24].

In 2002, the Agence Française de Sécurité Sanitaire des Aliments (AFSSA) granted approval for the consumption of *Odontella aurita*, citing its substantial equivalence to other edible seaweeds that had already been approved under EC Regulation 258/97 [25]. Since then, *O. aurita* has been officially designated as a Novel Food in the European Union (EU) [26]. According to the regulations set by the EU, the entire biomass of *O. aurita* can be utilized in certain food products, subject to maximum content limitations [27]. This particular species is one of the few commercially available options, even though it has not been officially recognized as safe for consumption by the United States Food and Drug Administration (US FDA) under the “generally recognized as safe” (GRAS) category [28]. Thus, it possesses the potential to be developed for feed, food, and functional material with high value-added properties [29,30]. The French company Innovalg has successfully cultivated this species on a large scale in raceway ponds and subsequently commercialized it as a dietary supplement [31]. The supplement is available in the form of capsules that contain dried cells. The species has not yet undergone human nutrition tests; however, it has demonstrated the potential to mitigate the risk of metabolic syndrome in mice that were fed a high-fat diet [21]. In addition, the lipophilic extract derived from this particular species has been recognized for its ability to mitigate the effects of skin aging, making it a popular choice as a cosmetic ingredient [29].

Acquiring new strains is of importance because the biological attributes of specific microalgae can vary depending on their habitat and strain, even within the same species [32]. Additionally, the regulations on Access and Benefit Sharing (ABS) under the Nagoya Protocol have recognized a variety of biomaterials, including microalgae, as valuable resources [33]. Per these regulations, any profits derived from these resources must be distributed among the resource providers. This has the potential to result in increased production expenses, thereby hindering the process of industrialization for the species [34]. Therefore, it can be argued that the exploration and cultivation of indigenous strains hold significant importance.

The objective of this research is to identify and analyze the indigenous *Odontella aurita* strain that was isolated from the coastal waters of Sonyang-myeon, Yangyang-gun, Gangwon-do, Republic of Korea. In addition, this study aims to determine the optimal culture conditions for each factor, including temperature, salinity, irradiance, and nutrients concentration, which have an impact on the growth of this species and analyze the composition of fatty acids and carotenoid pigments to examine its potential applications in various industries.

## 2. Results and Discussion

### 2.1. Morphological Identification of Strain OAOSH22

**Figure 1:**
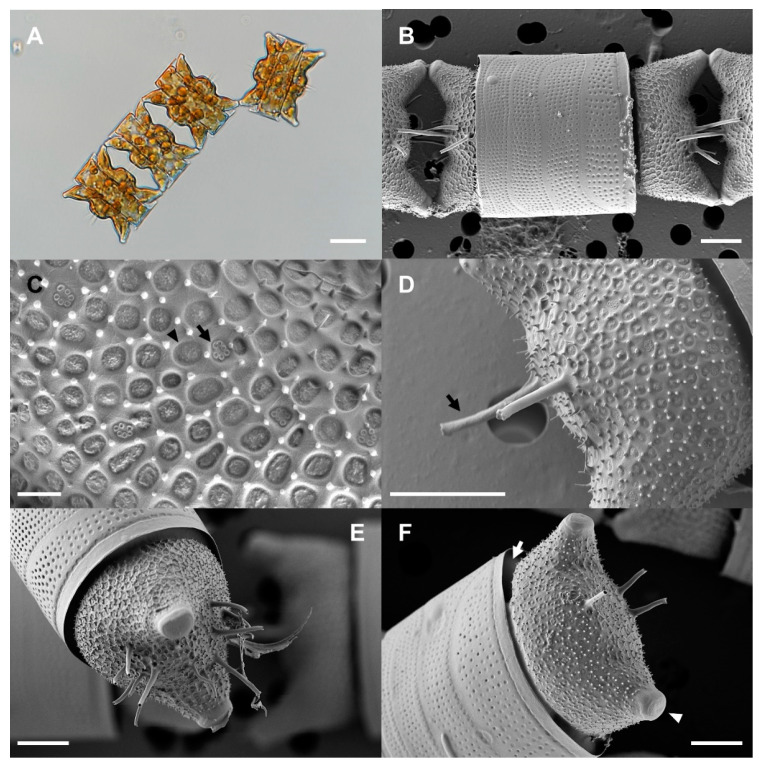
Light and scanning electron microscopy micrographs of *Odontella aurita* OAOSH22. (**A**) Cells form colonies with one or two horns connected. (**B**) External whole frustule view. (**C**) Detail of external areolae occluded by two types of velum (arrow and arrowhead). (**D**) Detail of external valve central area showing two labiate processes with spine-like external tubes (arrow). (**E**) Valve with 14 labiate processes. (**F**) Embedded valve in the girdle band (arrow) and two obtuse horns with ocelli at apices (arrowhead). Scale bars: (**A**) = 20 μm, (**B**,**D**–**F**) = 5 μm, and (**C**) = 1 μm.

Our morphological observations revealed that our isolate exhibited several features that are consistent with the characteristics of the genus *Odontella*. These features include bipolar valves, two elevations at the apices with rimmed ocelli at the summits, two types of pore occlusion, a distinct expanded hyaline valve margin with an upturned rim, rimoportulae located in the subcentral position, valvocopula extending beneath the flange, and chain formation [35]. The morphological characteristics of the isolate are outlined in detail below and illustrated in Figure 1. The cells were strongly silicified, with an apical axis measuring 25–51 μm (*n* = 16). Numerous small circular or elliptical chloroplasts were observed adhering to the cell wall (Figure 1A). The cells typically exhibited colony formation characterized by a zigzag pattern, with a single horn connecting them, or a linear colony formation, with both horns serving as points of connection (Figure 1A). Valves were more or less elliptical (bipolar), with two obtuse horns (elevations) and an ocellus at each pole. There was also a distinct convex area between the horns (Figure 1A,B,F; arrowhead). The valve mantle became increasingly constricted towards the edge and greatly curved outward from the edge again (Figure 1F; arrow). Valves were found to be embedded within the girdle band (Figure 1E,F). Two or more labiate processes (up to 14 observed) with spine-like external tubes were located in the central convex area of each valve (Figure 1D–F). The areolae were arranged radially from the center of the valve (11 in 10 μm, *n* = 7) and were occluded by two types of vela (Figure 1C; arrow and arrowhead). The surface of the valve exhibited a multitude of small spines (Figure 1C,D,F).

Microscopic observations revealed that strain OAOSH22 exhibited the characteristic morphological traits of *O. aurita* (Lyngbye) C.A. Agardh 1832 [35]. The size of *O. aurita* cells exhibits significant variability [16]. Therefore, it can be confusing to differentiate between *O. aurita* and other species that share similar morphological features, such as *O. obtusa* and *Hobaniella longicuris*. *O. obtusa* exhibits shorter and more obtuse horns, displaying greater inflection at the base and a lower elevation at the center of the valve compared to *O. aurita* [17]. In contrast to *O. aurita*, *H. longicruris* exhibits elongated and slender horns with minimal curvature at the base. Additionally, it possesses dome-shaped areolae [35].

### 2.2. Molecular Identification of Strain OAOSH22

**Figure 2:**
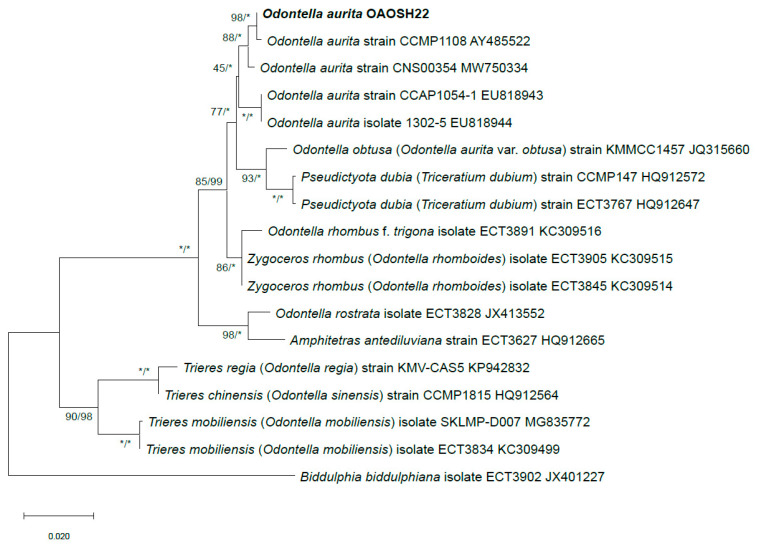
ML and BI phylogenetic tree of 18S rRNA gene from Eupodiscales species. The values on each node indicate ML bootstrap and Bayesian posterior probabilities (%), respectively. The asterisk (*) indicates 100.

The length of the trimmed and assembled 18S rDNA sequences for strain OAOSH22 was determined to be 1684 base pairs (bp). The sequences obtained as a result of this study have been submitted to GenBank under the accession number OP502635. A BLASTn search was conducted to determine the similarity of the 18S rDNA sequence of strain OAOSH22. The results revealed a high level of identity, with 99.6% similarity (query cover of 100% and E-value of 0), when compared to the 18S rDNA sequence of *Odontella aurita* (MW750334). Based on the BLASTn search results, we conducted phylogenetic analyses using maximum likelihood (ML), and Bayesian inference (BI) methods were used to confirm the taxonomic classification of strain OAOSH22 within the order Eupodiscales, which includes the genus *Odontella*. Strain OAOSH22 exhibited a close relationship with *O. aurita*, as evidenced by strong bootstrap values (ML bootstrap = 98% and BI posterior probabilities = 100%) (Figure 2). Finally, the strain OAOSH22 was identified as *Odontella aurita* via the analysis of morphological characteristics and sequencing data. The strain was deposited in the Korean Collection for Type Cultures (KCTC 15114BP).

Accurate identification of the microalgae species used for food is of utmost importance. Some microalgae have the ability to synthesize toxins, which can potentially lead to severe health complications [36]. Therefore, accurately identifying the microalgae and demonstrating that it belongs to a species that has been previously recognized as safe for consumption ensures that it is suitable for human consumption. In addition, accurate species identification holds significant importance from a quality control standpoint. Because each species of microalgae possesses distinct nutritional profiles and properties, precise identification of the target microalgae is crucial in ensuring that the final product achieves the intended nutritional content and properties [36]. In fact, the standardization of species identification is one of the research recommendations outlined in the Phycomorph European Guidelines for the Sustainable Aquaculture of Seaweeds [37]. In conclusion, precise species identification plays a crucial and indispensable role in ensuring the safety, quality, and nutritional value of microalgae during the industrialization process.

### 2.3. Optimization of Culture Conditions for Strain OAOSH22

**Figure 3:**
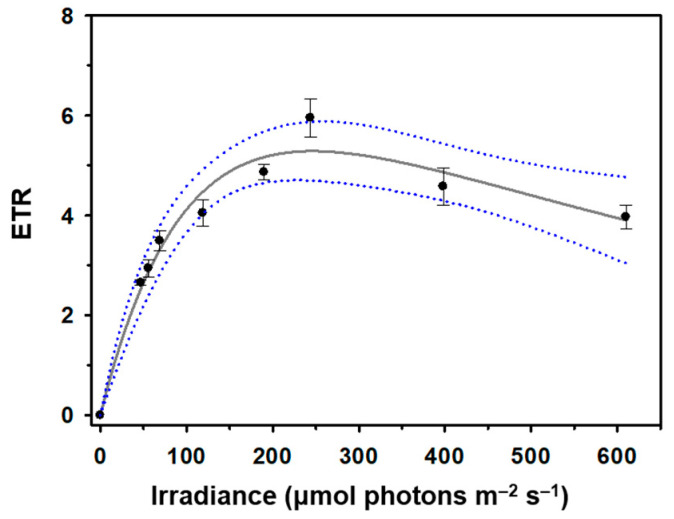
The rapid light-response curve of *Odontella aurita* OAOSH22. Solid lines indicate best fit according to model of Platt et al. [46], and blue dotted lines represent 95% confidence intervals (r^2^ = 0.96). Symbols and error bars represent the mean ± SE (*n* = 3).

**Figure 4:**
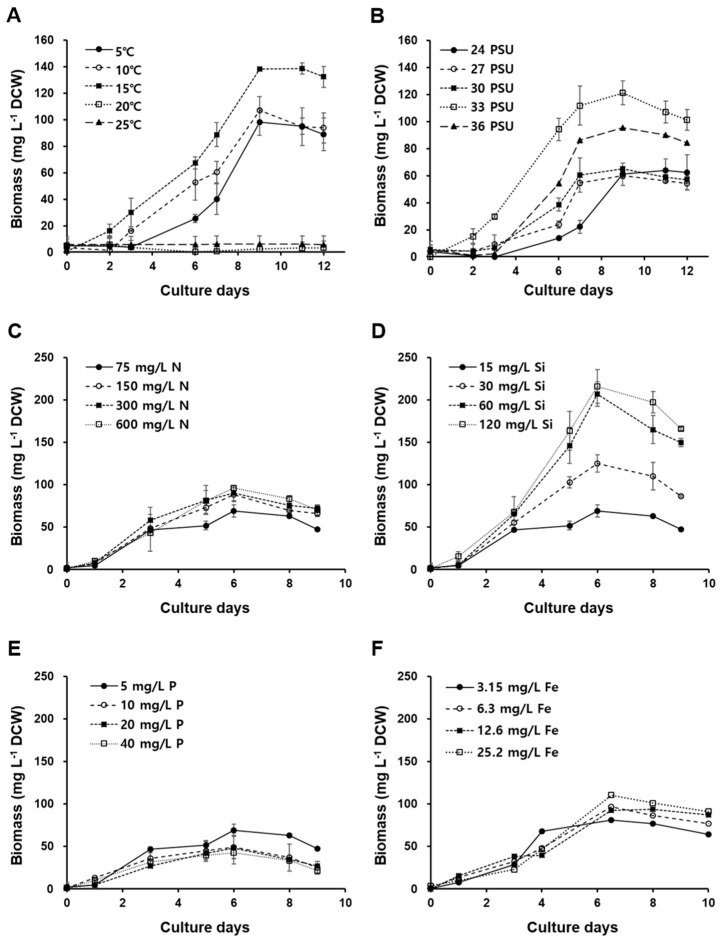
Growth curves of *Odontella aurita* OAOSH22 under different conditions of temperature (**A**), salinity (**B**), nitrate (**C**), silicate (**D**), phosphate (**E**), and iron (**F**). Symbols and error bars represent the mean ± SE (*n* = 3).

To assess the influence of various factors on the growth of *Odontella aurita* OAOSH22 and identify the optimal cultivation conditions for each factor, an analysis was conducted to examine the growth response under different conditions of irradiance, temperature, salinity, and nutrient concentration at the laboratory scale (Figure 3 and Figure 4). The growth rate and statistical analysis results for each treatment are depicted in Figure S1.

The optimal irradiance (*E_k_*) required to saturate photosynthesis in *O. aurita* OAOSH22 was determined via the rapid light curve method to be 76.5 μmol photons m^−2^ s^−1^. Furthermore, the *ETR_max_* was determined to be 5.29 (Figure 3). The growth of microalgae and biomass production are more significantly influenced by suitable irradiance rather than nutrient availability, as supported by previous studies [38,39]. At higher light intensities, where saturation occurs, additional illumination does not enhance the rate of photosynthesis. When microalgae are exposed to excessively intense light, it can result in photo-oxidative damage to the photosynthetic machinery via the generation of singlet oxygen. This subsequently reduces the efficiency and speed of photosynthesis, a phenomenon known as photoinhibition [40,41]. Furthermore, low levels of irradiance can hinder growth rates. Various studies have shown that certain species of microalgae are capable of achieving their highest growth rates when exposed to irradiances below 100 μmol photons m^−2^ s^−1^ [42]. Additionally, it has been observed that photoinhibition can occur even at irradiance levels ranging from 100 to 200 μmol photons m^−2^ s^−1^, which is significantly lower than the typical intensity of sunlight [43,44]. The results of this investigation showed similarities to the findings of previous studies. However, irradiances below 100 μmol photons m^−2^ s^−1^ may be considered suitable for laboratory-scale cultivation, as supported by the findings of this study. Conversely, when it comes to large-scale cultivations beyond the pilot scale, it may be necessary to increase light intensities in order to counteract the self-shading effects [45].

The growth curves of *O. aurita* OAOSH22 at temperatures of 5, 10, 15, 20, and 25 °C, respectively, are illustrated in Figure 4A. Biomass production reached its maximum value of 138.7 mg L^−1^ after a 9-day incubation period at a temperature of 15 °C (*p* < 0.05). However, no growth was observed at either 5 °C or 25 °C. Temperature is a critical determinant in the growth and development of microalgae [47]. Various aspects are influenced by it, including the growth rate, cell size, biochemical composition, and nutrient requirements [22]. *O. aurita* is a common species frequently found in temperate regions and present throughout the year. The species under consideration is classified as tychopelagic and is primarily distributed in coastal regions [48]. It primarily inhabits the seafloor during the summer and autumn, and can exert dominance in water columns from late winter to early spring [16,48]. This species was observed to thrive in a temperature range of −1.8 to approximately 26.0 °C, with the most favorable temperature for growth reported to be between −1.5 and 6.0 °C [49]. Martens [50] conducted a study at the Sylt–Rømø tidal basin, where it was found that a low temperature of −2 °C was the main factor responsible for the bloom of *O. aurita*. However, Baars [49] proposed that the species’ normal growth is best achieved at temperatures below 20 °C. In contrast, Pasquet et al. [51] conducted a study to investigate the impact of temperature on chlorophyll–fluorescent photosynthesis parameters and found that this particular species is capable of tolerating temperatures as high as 28 °C. Temperature critically affects photosynthesis efficiency via its impact on enzyme kinetics [52]. Lower temperatures impair enzymatic processes associated with photosynthesis, while moderate temperature elevations enhance respiratory rates [47]. In contrast, extreme temperatures suppress both metabolic and respiratory functions in microalgae [53]. Optimal microalgal growth is attained when energy production in the thylakoid membranes aligns with energy consumption in the Calvin cycle [47]. Environmental variations, especially temperature shifts, can disrupt this balance, leading to adjustments in the photosynthetic components, including altered structural dimensions and Rubisco activity [47]. At lower temperatures, carboxylase activity significantly diminishes. In contrast, at higher temperatures, some photosynthetic enzymes may cease to function. However, the tolerance of microalgae to these temperatures varies by species [47].

In order to achieve optimal growth of our strain, it is crucial to consider the salinity of the medium. The growth curves of *O. aurita* OAOSH22 at various salinities (24, 27, 30, 33, and 36 psu) are depicted in Figure 4B. Biomass production reached its peak at 138.2 mg L^−1^ on day 9 of the experiment at a salinity level of 33 psu (*p* < 0.05). Additionally, biomass production exhibited comparable levels within the range of 24 to 30 psu. There is limited existing research on the correlation between growth and salinity levels in *O. aurita*. However, McQuoid [54] found that low salinity levels below 15 psu could have a detrimental impact on the germination process of *O. aurita*. Salinity stress can significantly impact the growth and biochemical composition of microalgae. Indeed, salt stress has been identified as a primary factor affecting both the growth and biochemical composition of these organisms [55]. Different microalgae species exhibit preferences for specific salinity ranges, which are often associated with their natural habitats. This salinity affects osmotic and ionic balances, subsequently impacting growth, photosynthesis, and metabolite production [56]. For example, when *Chlorella vulgaris* is grown under varying salinity levels, it exhibits distinct metabolite profiles, characterized by variations in lipids, proteins, and carbohydrates [56]. Furthermore, extreme salinity can induce osmotic stress, potentially restricting the activity of ATP synthase and thus influencing crucial metabolic pathways [57].

In the present study, the biomass production of *O. aurita* OAOSH22 increased to approximately 100 mg L^−1^ (Figure 4C), which represents a 1.2-fold increase compared to the control, when the concentration of nitrate in the medium was doubled (*p* < 0.05). However, when the nitrate concentrations were doubled or higher, there was little to no increase in biomass production. Therefore, a concentration of 150 mg N L^−1^, which is twice the amount of nitrate found in the standard F/2 medium, appears to be adequate for the growth of *O. aurita* OAOSH22. Previous studies have consistently reported a strong correlation between the concentration of nitrate and the biomass of microalgae [58,59]. Similar findings have been observed in studies focusing on *O. aurita* [18,45,60]. However, Xia et al. [61] found that the biomass of *O. aurita* was produced at similar levels (approximately 4 g L^−1^) under both high (18 mM) and low (6 mM) nitrate concentrations when cultured at 100 μmol photons m^−2^ s^−1^. Additionally, they observed that biomass production increased approximately 1.5-fold under high nitrate concentrations compared to low concentrations when cultured at 300 μmol photons m^−2^ s^−1^. This observation demonstrates that providing sufficient nutrients alone may not guarantee optimal growth outcomes, as the fulfillment of basic physical environmental conditions is also crucial in determining growth effects.

In the present study, we observed that the using a silicate-enriched medium significantly enhanced the biomass production of *O. aurita* OAOSH22 (Figure 4D). The growth of *O. aurita* exhibited a significant increase with the rise in silicate concentration in the medium (*p* < 0.05). The maximum biomass of 216 mg L^−1^ was observed on the 6th day of culture when the silicate concentration was 8 times higher than that of the standard F/2 medium. Xia et al. [60] demonstrated that an increase in silicate concentration positively correlated with the biomass production of *O. aurita*. However, contrary to the findings of the present study, no significant difference in biomass production was observed across varying silicate concentrations (27.3–104.2 mg L^−1^). Silicates play a crucial role as vital nutrients in promoting diatom growth and are indispensable for the development of their cell walls composed of silica [62]. Therefore, the presence of silicates can have a substantial impact on the growth of diatoms [63]. When the availability of silicate is limited, a majority of diatoms experience disruptions in their cell cycles during the G1/S or G2/M phases, resulting in thinner frustules [62,64]. Additionally, the imposition of silicate restriction resulted in a reduction in the fucoxanthin content within *Phaeodactylum tricornutum* [65]. Conversely, it is imperative to appropriately adjust the concentration of silicate based on the target indicator material to be utilized, as research has shown that the restriction of silicate strongly promotes lipid accumulation in diatom cells [66].

In the case of phosphate, the biomass production of *O. aurita* exhibited no significant variation across different treatment concentrations, as depicted in Figure 4E. Phosphorus constitutes only 1% of the dry weight of microalgal cells; however, it plays a crucial role in limiting microalgal growth in natural ecosystems [22]. However, the impact of phosphorus on the growth of microalgae is relatively less significant compared to nitrogen. Additionally, it has been observed that beyond a certain concentration, phosphorus does not contribute to the growth and biomass production of microalgae [67,68]. Lu et al. [69] reported a negative correlation between phosphate concentration and biomass production in their study on the culture of *Nitzschia laevis*. As a result, it seems that there is no need to provide additional phosphate supply beyond the phosphate concentration present in the F/2 medium is not required for the growth of *O. aurita* OAOSH22.

Iron plays a crucial role in various metabolic processes that regulate photosynthesis via enzymatic reactions. It is a key component of cytochromes b and c, which function as electron transporters in both the photosynthetic and respiratory chain. This involvement of iron positively impacts the growth rate of diatoms [39,70]. The study conducted by Sahin et al. [12] demonstrated that *Nanofrustulum shiloi* exhibited 1.3- and 1.1-fold increases in response to an iron-rich environment. Contrarily, the limitation of iron frequently leads to an elevation in the silica composition of diatoms [71]. This, in turn, can cause a reduction in the concentration of silicate in the medium, ultimately resulting in the inhibition of diatom growth. In the present study, *O. aurita* OAOSH22 showed no significant variation in biomass production compared to the control group when given additional iron supplementation, (Figure 4F).

### 2.4. Carotenoid Content of Strain OAOSH22

**Figure 5:**
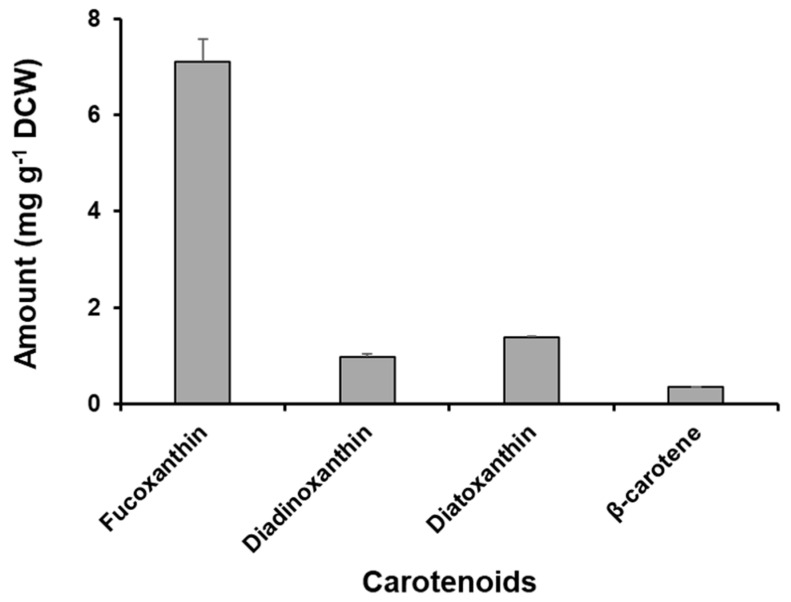
Concentrations of the major carotenoid pigments in *Odontella aurita* OAOSH22.

The concentration of major carotenoid pigments in *Odontella aurita* OAOSH22 is depicted in Figure 5. Additionally, the LC chromatogram can be found in Figure S2. The main carotenoid pigment found in the isolate obtained in this study was fucoxanthin, with a content of 7.10 ± 0.47 mg g^−1^ DCW. It was also found to contain a small amount of diadinoxanthin (0.98 ± 0.06 mg g^−1^ DCW) and diatoxanthin (1.37 ± 0.04 mg g^−1^ DCW).

Fucoxanthin, a xanthophyll pigment derived from carotenoids, is a naturally occurring pigment. According to Matsuno [72], it is estimated that this particular carotenoid contributes to over 10% of the overall carotenoid production in nature and holds the highest prevalence among carotenoids in marine ecosystems. In the context of fucoxanthin production, it is highly probable that commercially viable microalgae species would include diatoms (Bacillariophyta), Prymnesiales (Haptophyta), and Chrysophyceae (Ochrophyta) [73]. One notable chemotaxonomic characteristic of diatoms is their high concentration of fucoxanthin, which is also found in brown algae [74]. The fucoxanthin content in diatoms is approximately 1–6%, which is over 100 times greater than that found in brown algae [75]. Moreau et al. [29] conducted a study on the anticancer activity of fucoxanthin against bronchopulmonary cancer and epithelial cancer and reported that *O. aurita* is a significant source of fucoxanthin. Fucoxanthin exhibits health-promoting effects attributed to its potent antioxidant properties [76]. Additionally, it demonstrates anti-obesity, anti-diabetic, anti-cancer, anti-angiogenic, anti-inflammatory, anti-metastatic, and anti-Alzheimer’s disease activity [18,77]. Due to its diverse physiological activities, fucoxanthin has found extensive applications in the food, pharmaceutical, and cosmetic industries. It has gained significant attention as a functional material with anti-obesity properties [78]. Fucoxanthin has been scientifically proven to possess superior anti-cancer, anti-microbial, and free radical-scavenging properties compared to widely used compounds such as *β*-carotene and astaxanthin [79,80]. Based on the observed physiological activity, commercially available fucoxanthin-based products derived from microalgae, such as Fucovital and BrainPhyt^TM^, have been developed and marketed [81]. Fucovital is a health supplement developed by Algatechnologies. It is the first food additive to obtain US FDA approval in 2017 in recognition of its liver function improvement effect and is the first microalgae-derived fucoxanthin-containing product released on the market [22]. BrainPhyt^TM^ is a health supplement developed by Microphyt, France, and was listed on the US FDA’s New Dietary Ingredients (NDI) in 2019 [22]. It can help improve cognitive ability and short- and long-term memory by alleviating oxidative and inflammatory stress in the brain. Consequently, it is anticipated that there will be a rise in demand in the future [22].

Xia et al. [60] conducted a study on the fucoxanthin content of *O. aurita* and found that it reached up to 21.7 mg g^−1^ (dry weight). The actual content varied depending on the optimal culture conditions, specifically a light intensity of 100 μmol photons m^−2^ s^−1^ and a nitrate supply of 6 mM. This finding was significant, as it represented the highest reported fucoxanthin content in diatoms [82]. Although the fucoxanthin content observed in our study was lower than that reported in previous studies focusing on *O. aurita*, it was found to be comparable to or higher than the levels found in other species such as *Chaetoceros gracilis*, *Cylindrotheca closterium*, *Nitzschia laevis*, and *Phaeodactylum tricornutum* [68,83,84]. The composition of high-value-added substances can vary among different strains, even within the same species [32]. This variation is influenced by various culture conditions including light intensity [60], temperature [85], salinity [58], nutrient concentration [60], and culture media [86]. For instance, it has been observed that with an increase in light intensity, there is a corresponding increase in microalgal biomass [73]. However, it has also been noted that this increase in light intensity leads to a decrease in fucoxanthin production [87]. Light intensity exceeding 150 μmol photons m^−2^ s^−1^ has been found to induce the synthesis of photoprotective pigments, namely diadinoxanthin and diatoxanthin [88]. Therefore, further investigation into the optimal culture conditions is required in order to enhance the content of fucoxanthin.

### 2.5. Fatty Acids Composition of Strain OAOSH22

The composition of fatty acids in *Odontella aurita* OAOSH22 consisted of saturated fatty acids (SFAs) (42.5%), monounsaturated fatty acids (MUFAs) (37.8%), and polyunsaturated fatty acids (PUFAs) (19.7%). The main fatty acids synthesized by this strain were palmitoleic acid (C16:1, 36.4 ± 1.4%), palmitic acid (hexadecanoic acid, C16:0, 25.8 ± 1.0%), eicosapentaenoic acid (EPA, timnodonic acid, C20:5ω3, 17.7 ± 3.3%), and myristic acid (tetradecanoic acid, C14:0, 15.6 ± 1.2%) (Table 1).

The distribution of fatty acids exhibits significant variation among different microalgae taxa as well as within species. Diatoms are commonly known to possess a significant concentration of various fatty acids, including myristic acid (C14:0), palmitic acid (C16:0), palmitoleic acid (C16:1), stearic acid (C18:0), oleic acid (C18:1), and EPA (C20:5ω3) [13,89]. These fatty acids play a significant role in various industries, including food, pharmaceuticals, cosmeceuticals, aquaculture, and biofuel [77]. In particular, PUFAs, represented by EPA and docosahexaenoic acid (DHA), have garnered significant interest. PUFAs refers to unsaturated fatty acids containing 18 or more carbon and two or more double bonds [90]. PUFAs, such as omega-3 or omega-6 unsaturated fatty acids, play crucial roles in various physiological processes within the human body. However, these fatty acids are either not naturally synthesized (e.g., linoleic acid and α-linolenic acid) or are synthesized in limited quantities (e.g., EPA, DHA, and arachidonic acid). Consequently, it is necessary to obtain these PUFAs via dietary intake [91]. EPA offers a range of nutritional and health advantages, including its anti-inflammatory, anti-microbial, anti-cancer, vision and cardiovascular-protective, anti-Parkinsonian syndrome, and anti-Alzheimer’s disease effects [18,77].

*Odontella aurita* is a representative EPA-rich species among microalgae and is known to have an EPA content of more than 20% total fatty acids [18,45,51,92]. The fatty acid composition of *O. aurita* OAOSH22 was similar to that of previous studies, but the content of EPA was slightly lower, measuring at 17.7%. Several previous studies have documented that a deficiency of silicate in the growth medium stimulates lipid synthesis and leads to an increase in EPA levels [65,93]. Hence, it is hypothesized that the high concentration of silicate in the medium used in this study had a negative effect on the EPA content.

Currently, the primary source of EPA is derived from oily fish species such as salmon, mackerel, pilchard, herring, and trout [19]. However, diatoms present a promising alternative source of EPA, offering the advantage of meeting vegan dietary requirements [85]. In particular, the species *O. aurita* has already been commercially utilized for food in Europe, suggesting that it holds significant potential in the food and functional food industries [21,31]. In 2018, SAS Odontella, a French startup, introduced Solmon, a vegan food product that incorporates the extract of *Odontella aurita* to maintain the flavor profile of smoked salmon while also being a significant source of omega-3 fatty acids [94].

Myristic acid, which is the main fatty acid present in this strain, acts as a stabilizing agent for various proteins, including those involved in immune system function and anti-cancer properties [95]. Additionally, it has extensive applications in the beauty industry as a fragrance, surfactant, detergent, and emulsifier [96]. Palmitoleic acid has been documented to exhibit antibacterial properties [77], and has recently been suggested as a potential food ingredient for managing obesity [97].

## 3. Materials and Methods

### 3.1. Sample Collection and Isolation

A sample was collected from the coastal water at the port of Osan (38°5′25.51″ N, 128°39′53.36″ E) in Yangyang-gun, Gangwon-do, Republic of Korea, on 16 February 2022 by the Survey on Marine Bio-Resources. Cell isolation was conducted using the capillary method with a Pasteur pipette while being observed under an Eclipse Ti-U inverted microscope (Nikon, Tokyo, Japan). The isolated cells were subsequently transferred to a cell culture flask (SPL Life Sciences, Pocheon, Republic of Korea) containing F/2 medium supplemented with silicate (Sigma Aldrich Co., St. Louis, MO, USA) and a 0.2% antibiotic mixture (Penicillin-streptomycin-neomycin) (Sigma Aldrich Co.). To assess the ability of the isolated strain to grow on a solid medium, the monoculture strain was inoculated onto a 1% agar plate (Bacto Agar, BD Difco Ltd., Detroit, MI, USA) supplemented with F/2 medium. Culture strains cultivated in both liquid and solid media were periodically transferred to fresh medium at intervals of three weeks and two months, respectively. The culture strains were then incubated at a temperature of 17 °C, a 14:10 h light/dark cycle, and an irradiance of 40 μmol photons m^−2^ s^−1^.

### 3.2. Morphological Identification

The culture strain was harvested via centrifugation at 2100× *g* for 5 min and mixed with glycerol gelatin (Sigma Aldrich Co.) to be mounted on a slide. The mixed sample was placed dropwise on a glass slide and fixed in position with a coverslip. Finally, the margin of the coverslip was sealed with CoverGrip coverslip sealant (Biotium, Hayward, CA, USA). The slide was examined using a Nikon Eclipse Ni light microscope. For scanning electron microscopy (SEM), the cultured strain was fixed in 5% Lugol’s solution, filtered through a polycarbonate membrane with a pore size of 3 μm and a diameter of 25 mm (Advantec, Tokyo, Japan), and washed three times with sterile distilled water. The membrane was dehydrated in a graded series of ethanol (10%, 30%, 50%, 70%, 90%, and 100%) and finally dried using tetramethylsilane (Sigma Aldrich Co.). The membrane was mounted on a stub and sputter-coated with gold using an MC1000 ion sputter (Hitachi, Tokyo, Japan). The cells and surface morphology were observed using a high-resolution Zeiss Sigma 500 VP field-emission scanning electron microscope (FE-SEM, Carl Zeiss, Oberkochen, Germany).

### 3.3. Molecular Identification

The culture medium, which contained the strain, was transferred into a 50 mL conical tube and subjected to centrifugation at a speed of 5370× *g* for a duration of 5 min. The supernatant was subsequently removed. Genomic DNA extraction was performed using the DNeasy PowerSoil Pro Kit (Qiagen Inc., Hilden, Germany) according to the manufacturer’s instructions. Polymerase chain reaction (PCR) amplification was conducted using the Diatom9F [98]/EukBR [99] primer pairs in order to amplify the 18S rRNA sequence. PCR analysis was conducted following the protocol outlined by Raja et al. [100]. The PCR product underwent purification using ExoSAP-IT Express PCR Product Cleanup Reagent (Thermo Fisher Scientific, MA, USA) and was subsequently sequenced by Cosmogenetech Co., Ltd. (Seoul, Republic of Korea). The sequence underwent trimming, assembly, and alignment using Geneious Prime v.2022.2.2 (Biomatters Ltd., Auckland, New Zealand). The data set of 18S rRNA sequences was compiled, comprising the genetic sequences of 16 species belonging to the Eupodiscales order, as retrieved from GenBank. *Biddulphia biddulphiana* (JX401227) was utilized as an outgroup. Phylogenetic analyses were performed using maximum likelihood (ML) and Bayesian inference (BI) methods. Randomized Axelerated Maximum Likelihood (RAxML) v.8.2.10 [101] and MrBayes version 3.2.7 [102] were used for the ML and BI analyses, respectively. ML and BI analyses were conducted using the methodologies outlined by An et al. [103].

### 3.4. Determination of Optimal Culture Conditions

The isolate was cultured under various temperature, salinity, and nutrient conditions to determine the optimal culture conditions. The experimental conditions for water temperature, salinity, and irradiance were set based on previous research findings [38,49,51,54]. Additionally, the concentrations of each nutrient were set at levels that were 2, 4, and 8 times higher, respectively, than those found in the commonly used F/2 medium for culturing marine microalgae. To determine the optimal growth temperature, the temperature experiment compared growth at temperatures ranging from 5 to 25 °C. The salinity experiment, on the other hand, compared growth at salinities ranging from 24 to 36 psu using F/2 media that contained silicate. These experiments were conducted using a multi-thermo incubator (MTI-202B, Eyela, Tokyo, Japan). In addition, to confirm the growth characteristics based on nutrient concentrations, the isolate was cultured at 17 °C using a medium enriched with each nutrient (nitrate, silicate, phosphate, and iron) (Sigma Aldrich Co.). The concentration of each nutrient in the standard F/2 medium was set to the control level. Optimal conditions for each factor were determined via daily growth tests. Detailed experimental conditions for each factor are shown in Table 2. Samples for all tests, with the exception of the test aimed at determining optimal irradiance, were carried out by introducing 30 mL of medium containing the strain into a 25 cm^2^ cell culture flask. A dimmable LED panel was used as the light source for the cultivation process. Irradiance was measured using the HD2102.2 Portable Luxmeter Data Logger, which was equipped with LP471PAR Quantum Radiometric Probe (Delta OHM, Caselle, Padova, Italy).

To determine the optimal growth irradiance, the pulse amplitude modulation (PAM) fluorometry technique was used in this study. The PAM technique is commonly employed to assess parameters associated with the photosynthetic efficiency of microalgae using chlorophyll fluorescence quenching analysis [104]. RLCs obtained using the PAM technique offer comprehensive insights into the saturation characteristics of electron transport and the overall photosynthetic capacity of microalgal strains [105]. This information can be used to determine the optimal level of irradiance required for cultivating specific types of microalgae [106]. It can also be used to estimate the maximum productivity of the culture when provided with the optimal irradiance [107]. The determination of the light saturation coefficient (*E_k_*), which indicates the point at which photosynthesis reaches saturation, involves considering two factors: the maximum electron transport rate (*ETR_max_*) and the initial slope (*α*) of the RLC. The initial slope of a graph represents the quantum efficiency of photosynthetic electron transport [108]. *E_k_* can be regarded as the ideal irradiance level for the cultivation of microalgal strains [109]. A 3.0 mL quantity of the culture strain was placed in a DUAL-K25 quartz glass cuvette, which was supplied with the Dual-PAM and dark-adapted for 30 min before PAM measurement. Rapid light curve (RLC, ETR versus irradiance curve) was conducted at eight incremental irradiances (47, 56, 69, 119, 190, 244, 398, and 610 µmol photons m^−2^ s^−1^) of actinic light using the Dual-PAM-100 (Heinz Walz Gmbh, Effeltrich, Germany) equipped with an Optical Unit ED-101US/MD. The light curve was fitted according to the model of Platt et al. [46] to determine the maximum electron transport rate (*ETR_max_*), the initial slope of the curve (*α*), and the irradiance at which ETR saturation occurs (*E_k_*). Data processing was performed following the method described in Ralph and Gademann [105] using SigmaPlot v.12.3 (Systat Software Inc., San Jose, CA, USA).

### 3.5. Determination of Biomass

Biomass production was calculated based on the equation derived from the calibration curve between chlorophyll fluorescence (with an excitation wavelength of 440 nm and emission wavelength of 680 nm) and dry cell weight (DCW) (Figure S3). To obtain a calibration curve of chlorophyll fluorescence versus biomass weight, we measured the chlorophyll fluorescence of five pre-cultures of algae with different cell densities using a microplate reader (Synergy H1, BioTek, Winooski, VT, USA). Each sample was then placed in a pre-weighed tube and centrifuged at 8400× *g*. After the supernatant was removed, the sample was washed once with distilled water to remove salt and then centrifuged again. The cell pellet was lyophilized using a benchtop freeze dryer for 24 h (Freezezone 4.5, Labconco, Kansas City, MO, USA). Finally, the DCW was determined by subtracting the weight of the pre-weighed tube from the total weight of the tube containing the pellet.

### 3.6. Carotenoids Analysis

Carotenoid was analyzed using a slight modification of the method from Kang et al. [110]. To quantify the carotenoid content, algal biomass concentration was calculated using a pre-determined conversion equation mentioned earlier. Subsequently, algal cells were collected by centrifugation at 33,600× *g* for 2 min. The supernatant was then removed, and the pigments were extracted from the cells using 100% methanol and algal cells disrupted via ultrasonic water bath (DAIHAN Scientific, Republic of Korea) for 3 min at 60 °C. The resulting supernatant was then filtered through a 0.2 μm PTFE membrane filter (Millipore, Billerica, MA, USA). Carotenoid pigments were analyzed using an Agilent 1260 Infinity HPLC system (Agilent, Waldbronn, Germany) equipped with a Spherisorb 5.0 μm ODS1 4.6 × 250 mm cartridge column (Waters, St. Louis, MO, USA) at 40 °C. Chromatograms were identified by comparing them to carotenoid standards including fucoxanthin (Sigma Aldrich, St. Louis, MO, USA), diadinoxanthin, diatoxanthin, and β-carotene (DHI, Hørsholm, Denmark), and the concentration of each pigment was calculated using the peak area of the standard pigments.

### 3.7. Fatty Acid Analysis

Cells were harvested via centrifugation at 16,500× *g* for fatty acid analysis. After + as much supernatant as possible was removed, the pellets were lyophilized via freeze-drying at −110 °C under a vacuum for 24 h. Fatty acid extraction was performed following the methods described by Garces and Mancha [111]. The fatty acid composition was analyzed using a 7890A gas chromatograph (Agilent, Wilmington, DE, USA) equipped with a flame ionization detector (280 °C, H2 35 mL min^−1^, air 350 mL min^−1^, He 30 mL min^−1^) and a DB-23 column (60 mm × 0.25 mm × 0.25 μm film thickness; Agilent). The initial GC oven temperature was set at 80 °C and maintained for 3 min. The temperature was ramped up at a rate of 15 °C min^−1^ to 200 °C and held for 8 min. It was then ramped at a rate of 1 °C min^−1^ to 215 °C and held for 8 min. After that, it was ramped at a rate of 2 °C min^−1^ to 250 °C and held for 5 min. Finally, it was ramped at a rate of 50 °C min^−1^ to 80 °C and held for 3 min. The sample (2 μL) was injected with a split ratio of 10:1. The injector and detector temperatures were set at 250 °C and 280 °C, respectively. The identification of fatty acids was performed by comparing their retention time to the retention time of standards (Supelco 37-component FAME mix; Supelco, Bellefonte, PA, USA) and an internal standard (pentadecanoic acid; Sigma Aldrich Co.). Fatty acid analysis was conducted at the National Instrumentation Center for Environmental Management (NICEM) at Seoul National University in the Republic of Korea.

### 3.8. Statistics Analysis

All experimental procedures were conducted in triplicate. First, the data were subjected to analysis in order to evaluate its normality through the utilization of the Shapiro–Wilk test. Once the normality of the data was confirmed, the mean differences between treatments were examined via one-way analysis of variance (ANOVA) with Tukey’s post hoc test (*p* < 0.05) using the SPSS v.14.0 software (SPSS Inc., Chicago, IL, USA). The mean and standard deviations are reported, and distinct letters are used to indicate a statistically significant difference at a significance level of *p* < 0.05.

## 4. Conclusions

In the present study, we obtained an indigenous strain of *Odontella aurita* OAOSH22 and conducted an analysis of its fundamental characteristics and optimal culture conditions. As observed in previous studies on this species, the current isolate also exhibited elevated levels of fucoxanthin and EPA. This particular species has obtained certification and has been utilized as a cosmetic ingredient not only in Korea but also in numerous other countries. Furthermore, it possesses significant potential as a material for food and health functional products. The composition of high-value-added substances is subject to the influence of diverse cultural conditions, including light intensity, temperature, salinity, nutrient concentration, and culture media. Therefore, additional investigation is required to enhance the synthesis of valuable compounds. In addition, in order to establish optimal conditions for large-scale cultivation in an industrial environment, follow-up research to reduce cultivation costs, such as exploring alternatives to expensive media, must also be conducted.

## Figures and Tables

**Table 1:** Fatty acid composition (% total fatty acids) of *Odontella aurita* OAOSH22.

Fatty Acids			Amount (mg g^−1^ DCW)	Composition (%)
SFA	Myristic acid	C14:0	9.65 ± 0.23	15.61 ± 1.20
	Palmitic acid	C16:0	15.96 ± 0.27	25.76 ± 0.95
	Stearic acid	C18:0	0.68 ± 0.02	1.10 ± 0.09
MUFA	Palmitoleic acid	C16:1n7	22.52 ± 0.36	36.34 ± 1.36
	Oleic acid	C18:1n9	0.93 ± 0.02	1.50 ± 0.05
PUFA	Linoleic acid	C18:2n6 cis	0.75 ± 0.03	1.21 ± 0.01
	Gamma-linolenic acid (GLA)	C18:3n6	0.30 ± 0.03	0.48 ± 0.03
	Arachidonic acid (AA)	C20:4n6	0.22 ± 0.06	0.33 ± 0.09
	Eicosapentaenoic acid (EPA)	C20:5n3	11.07 ± 2.63	17.66 ± 3.28

**Table 2:** Experimental conditions for determining optimal cultivation conditions for each factor. The minimum condition for each nutrient utilized in the experiments is its presence in a standard F/2 medium.

	Experimental Conditions
Temperature (°C)	5, 10, 15, 20, 25
Salinity (psu)	24, 27, 30, 33, 36
Nutrients (mg L^−1^)	
Nitrate (NaNO_3_)	75, 150, 300, 600
Silicate (Na_2_SiO_3_•9H_2_O)	15, 30, 60, 120
Phosphate (NaH_2_PO_4_•H_2_O)	5, 10, 20, 40
Iron (FeCl_3_•6H_2_O)	3.15, 6.3, 12.6, 25.2

## Data Availability

Data are contained within the article.

## Supplementary Materials

The following supporting information can be downloaded at: https://www.mdpi.com/article/10.3390/md21110563/s1, Figure S1: Relative growth rates of *Odontella aurita* OAOSH22 under different conditions of temperature (a), salinity (b), nitrate (c), silicate (d), phosphate (e), and iron (f). Symbols and error bars represent the mean ± SE (*n* = 3). The same letter indicates homologous groups as recognized via the Tukey HSD test at *p* = 0.05. Figure S2: The chromatogram, acquired via high-performance liquid chromatography with photodiode array detection (HPLC-PDA), of the carotenoid extract derived from *Odontella aurita* OAOSH22. Figure S3: Calibration curve for *Odontella aurita* OAOSH22 based on fluorescence excitation of chlorophyll.
