# Supplementary figures and images for: Description and Characterization of the Odontella aurita OAOSH22, a Marine Diatom Rich in Eicosapentaenoic Acid and Fucoxanthin, Isolated from Osan Harbor, Korea
Source: Mar Drugs. 2023 Oct 27;21(11):563. doi: 10.3390/md21110563 (PMC10671887; doi:10.3390/md21110563)

A

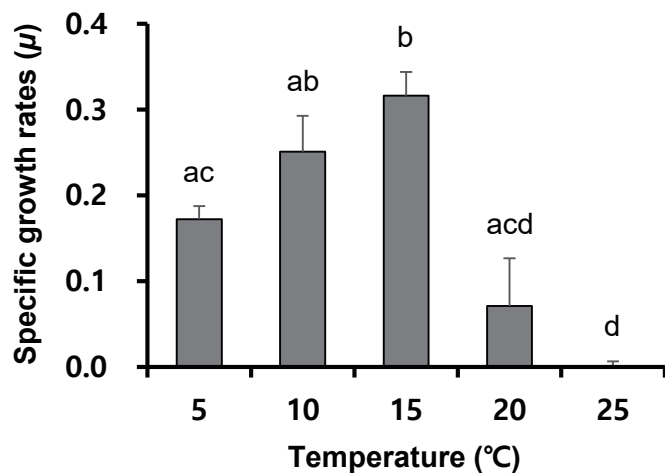

B

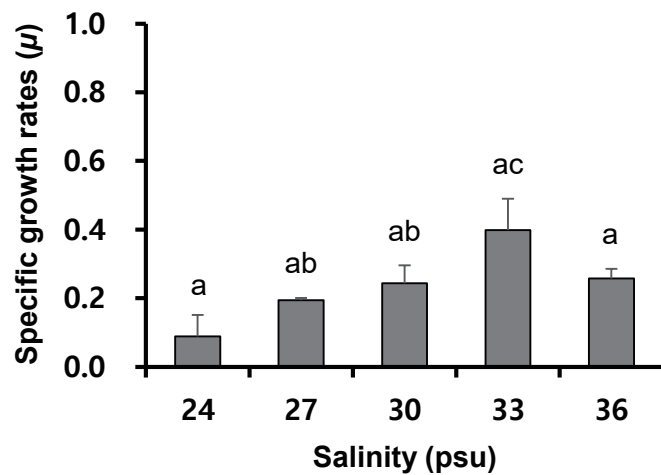

C

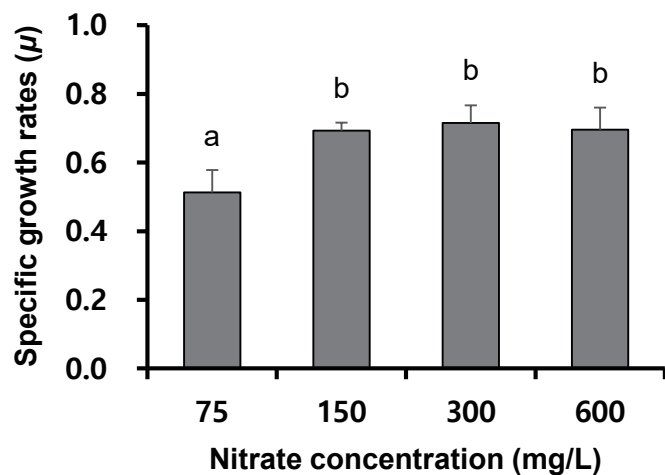

D

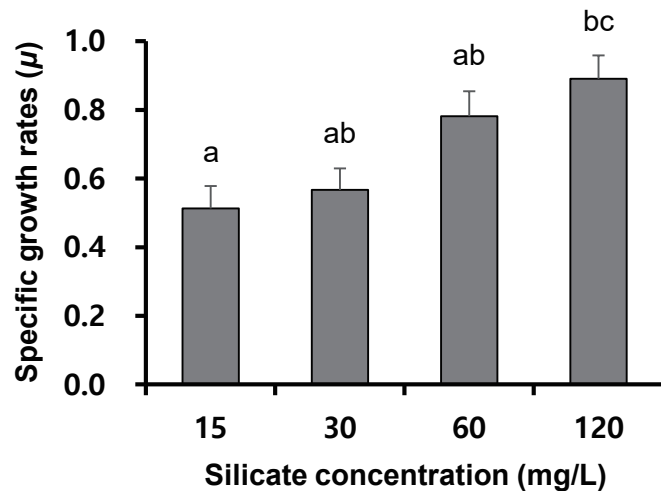

E

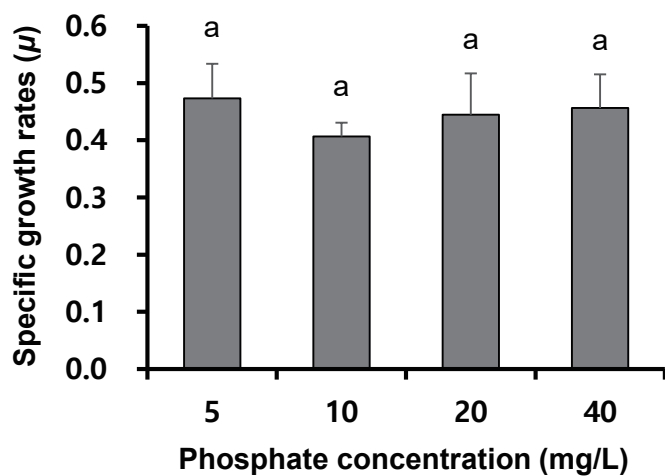

F

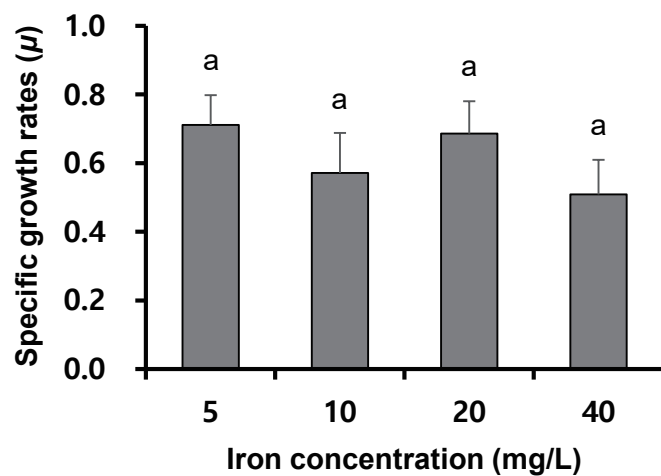

Supplement: Supplementary file 1 [file marinedrugs-21-00563-s001.zip › Figure S1.pdf]

DAD1 B, Sig=445,4 Ref=off (1BE-1501.D)

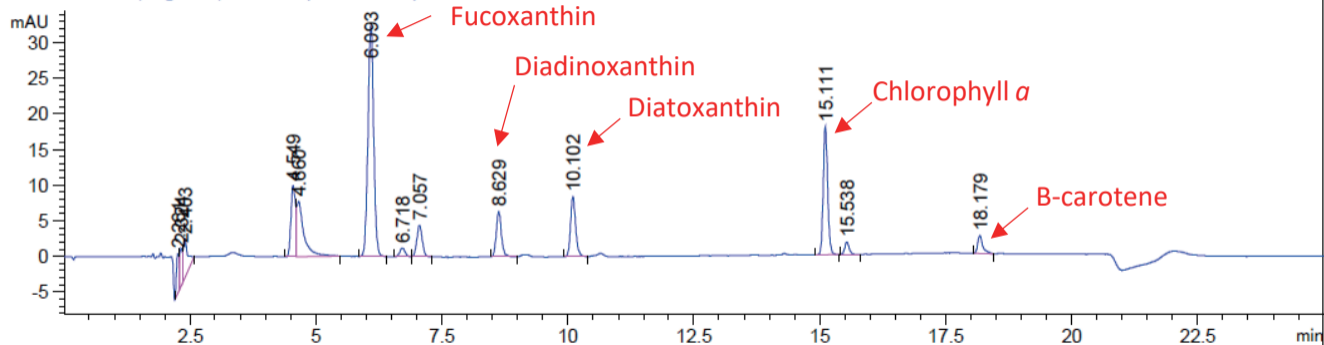

Supplement: Supplementary file 1 [file marinedrugs-21-00563-s001.zip › Figure S2.pdf]

## Fluorescence

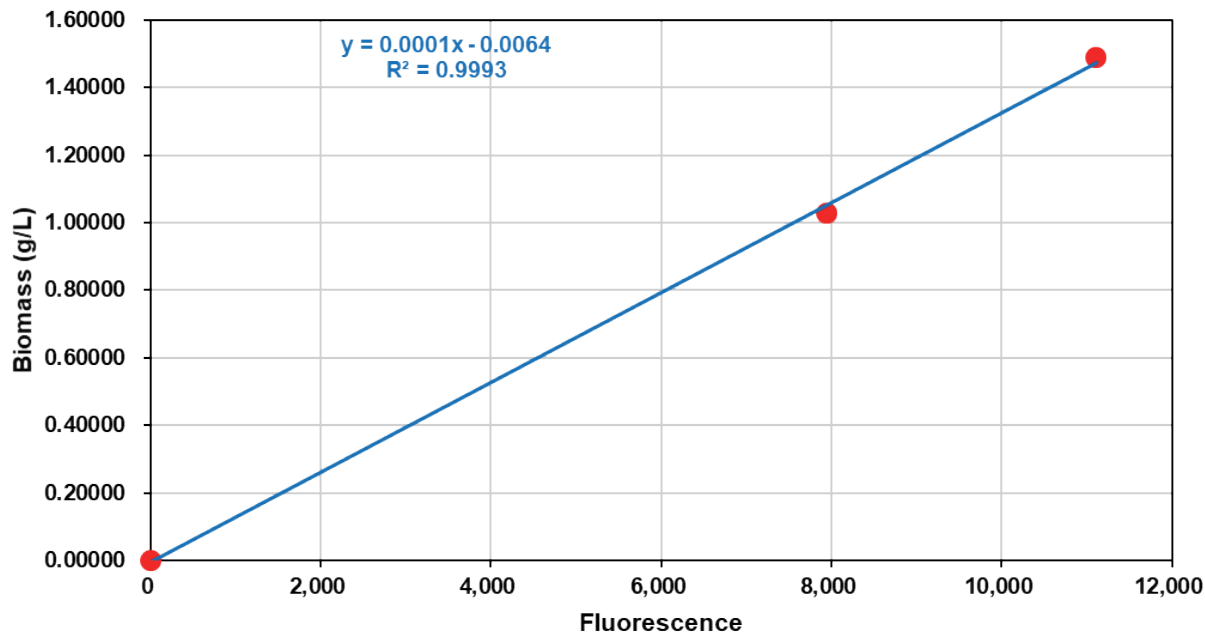

Fluorescence

$$y = 0.0001x - 0.0064$$

$$R^2 = 0.9993$$

Supplement: Supplementary file 1 [file marinedrugs-21-00563-s001.zip › Figure S3.pdf]
